# Direct vs. redirected admission of critically ill children to PICU after interfacility transfer: a retrospective cohort study

**DOI:** 10.3389/fped.2024.1307565

**Published:** 2024-02-16

**Authors:** C. Halgren, G. M. Annich, C. Maratta

**Affiliations:** ^1^ Department of Critical Care, Hospital for Sick Children, Toronto, ON, Canada; ^2^ Interdepartmental Division of Critical Care Medicine, University of Toronto, Toronto, ON, Canada; ^3^ Institute of Health Policy, Management and Evaluation, University of Toronto, Toronto, ON, Canada; ^4^ Department of Paediatrics, University of Toronto, Toronto, ON, Canada

**Keywords:** pediatric transport, critical care transport, interfacility transport, PICU, remoteness

## Abstract

**Background:**

Critically ill children must often be transported long distances for access to critical care resources in Canada. This study aims to describe and compare characteristics and outcomes in patients presenting in the community and requiring inter-facility transport and admission to a Pediatric Intensive Care Unit (PICU).

**Methods:**

This is a retrospective cohort study of children admitted to the ICU at the Hospital for Sick Children from 2016 to 2019 after inter-facility transport. Characteristics and outcomes were compared between children admitted to the PICU within 24 h from their initial critical care transport request, and children admitted after initial redirection to a non-ICU care setting, 24–72 h from request. The primary outcome was severity of illness at PICU admission. Secondary outcomes included duration of mechanical ventilation, organ dysfunction, PICU length of stay and mortality.

**Results:**

A total of 2,730 patients were admitted after inter-facility transport to either the medical/surgical or cardiac ICU within 72 h of initial critical care transport request. Of these children, 2,559 (94%) were admitted within 24 h and 171 (6%) were admitted between 24 and 72 h. Children admitted after initial redirection were younger and residing in more rural centers. Children who were initially redirected had lower severity of illness (PRISM-IV median score 3 vs. 5, *p* = 0.047) and lower risk of mortality.

**Interpretation:**

Initial redirection to a non-ICU care setting rather than directly admitting to the PICU did not result in increased severity of illness or mortality. This study highlights the need to better understand which factors influence disposition decision-making at the time of initial transport request. Further research should focus on the impact of transport factors on clinical outcomes after PICU admission.

## 1 Introduction

Treatment at dedicated pediatric tertiary care centers, in addition to transport by specialized pediatric transport teams, have improved outcomes and survival among critically ill children (1–4). This population of children who undergo interfacility transport prior to pediatric intensive care unit (PICU) admission has been demonstrated to have increased severity of illness, increased resource utilization in ICU and increased mortality, when compared to children directly admitted from within a pediatric tertiary care institution (5–9).

Given the expansive geography of Canada, critically ill children must often be transported long distances for access to critical care resources. Remoteness is associated with both increased PICU and hospital length of stay, increased difficulty in accessing tertiary ICU care (7), and distance travelled is associated with increased risk of mortality in Canada (7, 10). There is potential risk associated with transport over long distance, a common occurrence in Canada (11, 12), which may be in part reason for the push towards local stabilization and goal-directed care, and away from the “scoop and run” paradigm (13). There is also increasing recognition that some children referred to tertiary center PICUs may not require critical care and could potentially be cared for and monitored in other care areas and institutions (11–16). In a study describing transport in Ontario, Canada, approximately half of children for whom transport and access to PICU services were requested were not ultimately admitted to an intensive care (17). Little is known about patient characteristics which may determine the possibility of safe observation and delayed admission to ICU after initial transport and reassessment. To date, no study has examined the characteristics and outcomes of critically ill patients admitted to the PICU after initial redirection to a non-ICU care setting, or prolonged local stabilization. This study aims to describe and compare characteristics and outcomes in patients presenting in the community and requiring inter-facility transport and admission to ICU.

## 2 Methods

### 2.1 Design and population

This was a retrospective cohort study of children aged 0–18 year old admitted to the Critical Care Unit at the Hospital for Sick Children, after inter-facility transport.

### 2.2 Setting

The Hospital for Sick Children is a quaternary pediatric care center in Canada, with both a 21-bed medical and surgical ICU with approximately 1,300 annual admissions, and a 21-bed cardiac ICU, with approximately 800 admissions annually. The critical care transport telephone consultation line is a service facilitated by CritiCall, which is a provincial healthcare organization providing referring physicians and centers with direct support and access to acute care resources. It is an acute care consultation and transport request line that runs 24 h a day, 7 days a week. Telephone calls from community providers to CritiCall are usually directed to one of four PICUs in Ontario, based on the location of the referring center. The Hospital for Sick Children receives approximately 1,200 requests for critical care access and transport annually. When a critical care transport call is received, demographic information is obtained, and clinical assessment is made regarding need for acute care. Thereafter, transport is organized and/or advice is provided over the phone by an ICU fellow and attending. Once a decision to transfer is made, either the in-house specialized paediatric transport team is dispatched if available, or a provincially run transport team facilitates the transport. The patient is subsequently transported to a specified location, most often to a tertiary or quaternary pediatric care center. Initial disposition determination is made during phone consultation and may result in direct admission to the ICU, redirection (to a hospital ward or the emergency department), deferral to another ICU, or advice provision only. The decision regarding disposition is made during the telephone consultation with the referring physician, based on patient characteristics, presenting history, suspected diagnosis, clinical course and evolution, and vital signs. No pre-specified criteria or guidelines dictate disposition, and the ultimate decision is at the discretion of the ICU physician taking the telephone transfer request. The institutionally run paediatric transport team is comprised of a pediatric nurse and respiratory therapist with specialized training in advanced neonatal and pediatric care.

### 2.3 Participants

All children aged 0–18 years for whom transport was requested between January 01, 2016 to December 31, 2019 who were transported to the Hospital for Sick Children and who were admitted to the PICU within 72 h were included in the cohort. Patients over 18 years of age, who were premature (less than 36 weeks gestational age) were excluded, as were patients requiring elective inter-provincial transport. The cohort was comprised of patients admitted to the PICU within 24 h from their initial transport telephone request, and patients admitted between 24 and 72 h from initial transport request. Patients admitted to ICU 24–72 h from initial request were either initially admitted elsewhere within the institution after transport, or were kept in their community centre after the initial phone call.

### 2.4 Data sources and variables

Data were extracted from the institution's transport telephone call database. This database includes basic demographic and referring hospital data, principal diagnosis, and initial disposition decision. For patients who were subsequently admitted to institution's critical care unit, clinical data were extracted from a local database and linked to transport data. Patient data were deterministically linked when hospital number was available, and were probabilistically linked using age, sex, and ICU date of admission when hospital number was missing. The following data elements were collected: age, sex, primary diagnosis, admission source, location within referral hospital, referring hospital characteristics, severity-of-illness score [Pediatric Risk of Mortality IV (PRISM-IV)] on admission, length of PICU stay, use of mechanical ventilation or non-invasive ventilation during admission, organ dysfunction [maximum Pediatric Logistic Organ Dysfunction-2 score (PELOD-2)], and mortality. Rural status of referring center was determined using definitions taken from Statistics Canada. A rural/small center is defined as an area with a population between 1,000 and 29,999, a medium center is an area with a population between 30,000 and 99,999, and a large urban center has a population of 100,000 or more^1^. Transport distances were one way and calculated using an online tool by inputting referral hospital address and measuring distance by land and by crow (i.e., for air transport) to the Hospital for Sick Children as the reference destination (18). The primary outcome was severity of illness on admission, as defined by the PRISM-IV score. PRISM-IV is a validated composite score calculated using 17 physiologic variables collected on PICU admission to predict the risk of mortality (19). Secondary outcomes included the need for and duration of mechanical ventilation during admission, severity of organ dysfunction, length of stay in the PICU and PICU mortality.

### 2.5 Statistical analysis

Descriptive statistics were used to summarize the demographic characteristics of the study population. Continuous data were expressed as means and standard deviations for normally distributed data, and medians and interquartile ranges (IQRs) for non-parametric data. We compared medians using the Mann–Whitney *U* rank-sum test. Categorical data were summarized as counts and proportions and compared using the *χ*^2^ test. Adjustment for severity of illness or other potential confounders was not performed for statistical comparisons using the *χ*^2^ test or the Mann–Whitney *U* rank-sum test. Subgroup analysis was performed in the subset of patients admitted to the cardiac intensive care unit. Data were statistically analyzed with Stata (Stata/IC 15.1) and R Studio. Statistical significance was considered at a *p* value less than 0.05. Missing outcome or covariate data were not imputed and are reported as missing in Tables 1, 2 and detailed in the footnotes. Ethics approval was obtained from the Hospital for Sick Children Research Ethics Board (no. 1000075972).

**Table 1:** Characteristics of patients admitted to the ICU after inter-facility transport.

Characteristic	Total, *n* = 2,730	Admission <24 h from call, *n* = 2,559	Admission within 24 h–72 h from call, *n* = 171
Sex, *n* (%)			
Male	1,525 (55.9)	1,432 (56)	93 (54.4)
Female	1,204 (44.1)	1,126 (44)	78 (45.6)
Missing	1 (0)	1 (0)	0
Age in years, (IQR)	2.2 (0.2–10)	2.4 (0.2–10)	0.6 (0.03–5.7)
Primary Diagnosis, *n* (%)			
Cardiac/Circulation	786 (28.8)	719 (28.1)	67 (39.2)
Cardiac Arrest/VSA	36 (1.3)	35 (1.4)	1 (0.6)
CDH	39 (1.4)	38 (1.5)	1 (0.6)
DKA	217 (8)	214 (8.4)	3 (1.8)
Ingestion/Overdose	80 (2.9)	77 (3)	3 (1.8)
Neurologic	510 (18.7)	479 (18.7)	31 (18.1)
Respiratory	816 (29.9)	768 (30)	48 (28.1)
Sepsis/Infection	13 (0.5)	12 (0.5)	1 (0.6)
Trauma/Drowning/Burn	113 (4.2)	107 (4.2)	6 (3.5)
Other	115 (4.2)	105 (4.1)	10 (5.9)
Missing	5 (0.2)	5 (0.2)	0
Urban Centre^a^, *n* (%)			
Rural or small	238 (8.7)	207 (8.1)	31 (18.1)
Medium	111 (4.1)	111 (4.3)	0
Large	2,381 (87.2)	2,241 (87.6)	149 (81.9)
Distance^b^, (minimum, maximum)	30.3 (10.9–353.9)	30.3 (10.9–353.9)	41.5 (25.6–79.3)

CDH, congenital diaphragmatic hernia; DKA, diabetic ketoacidosis; VSA, vital signs absent; IQR, interquartile range.

All continuous data are presented in medians and interquartile ranges, unless otherwise specified.

^a^ Rural/Small center: any area outside of a population center or a population between <1,000 and 29,999. Medium center: population between 30,000 and 99,999. Large center: population of 100,000 or more.

^b^ Distance from referral center to the institution in kilometers (as the crow flies). The IQR for 30.3 (18.8, 66.8), for 30.3 was (17.3, 70.6) and for 41.5 was (41.5, 41.5).

**Table 2:** Outcomes of patients admitted to the ICU after inter-facility transport.

Outcome	Total, *n* = 2,730	Admission <24 h from call, *n* = 2,559	Admission 24h-72 h from call, *n* = 171	*p*-value
Admission PRISM-IV score, (IQR)	5 (1–9)	5 (1–9)	3 (0–8)	0.047
Need for non-invasive mechanical ventilation, *n* (%)^a^	535 (19.6)	493 (19.3)	42 (24.6)	0.09
Length of non-invasive ventilation, (IQR)	2 (1–4)	2 (1–4)	2 (1–4)	0.99
Need for invasive mechanical ventilation, *n* (%)^a^	1,315 (48.2)	1,237 (48.3)	78 (45.6)	0.49
Length of invasive mechanical ventilation in days, (IQR)	3 (2–7)	3 (2–7)	4 (2–8)	0.33
Maximum PELOD-2 score during admission, (IQR)	11 (2–20)	11 (2–20)	11 (3–20)	0.91
Length of stay in days, (IQR)	3 (2–6)	3 (2–6)	3 (2–7)	0.26
Mortality, *n* (%)^a^	151 (5.5)	148 (5.8)	3 (1.8)	0.026

PRISM-IV, Pediatric Risk of Mortality IV; PELOD-2, Pediatric Logistic Organ Dysfunction-2; IQR, interquartile range. Missing data: 569 patients had missing PRISM-IV data (20%), 346 patients had missing PELOD scores (13%), 333 patients had missing data for length of stay (12%). Complete data were present for 2,161 (79%) for the primary outcome.

^a^ These factors were compared using the chi-square test of proportions. All other outcomes were compared using the Mann–Whitney *U* rank-sum test. All continuous data are presented as medians and interquartile ranges.

## 3 Results

**Figure 1:**
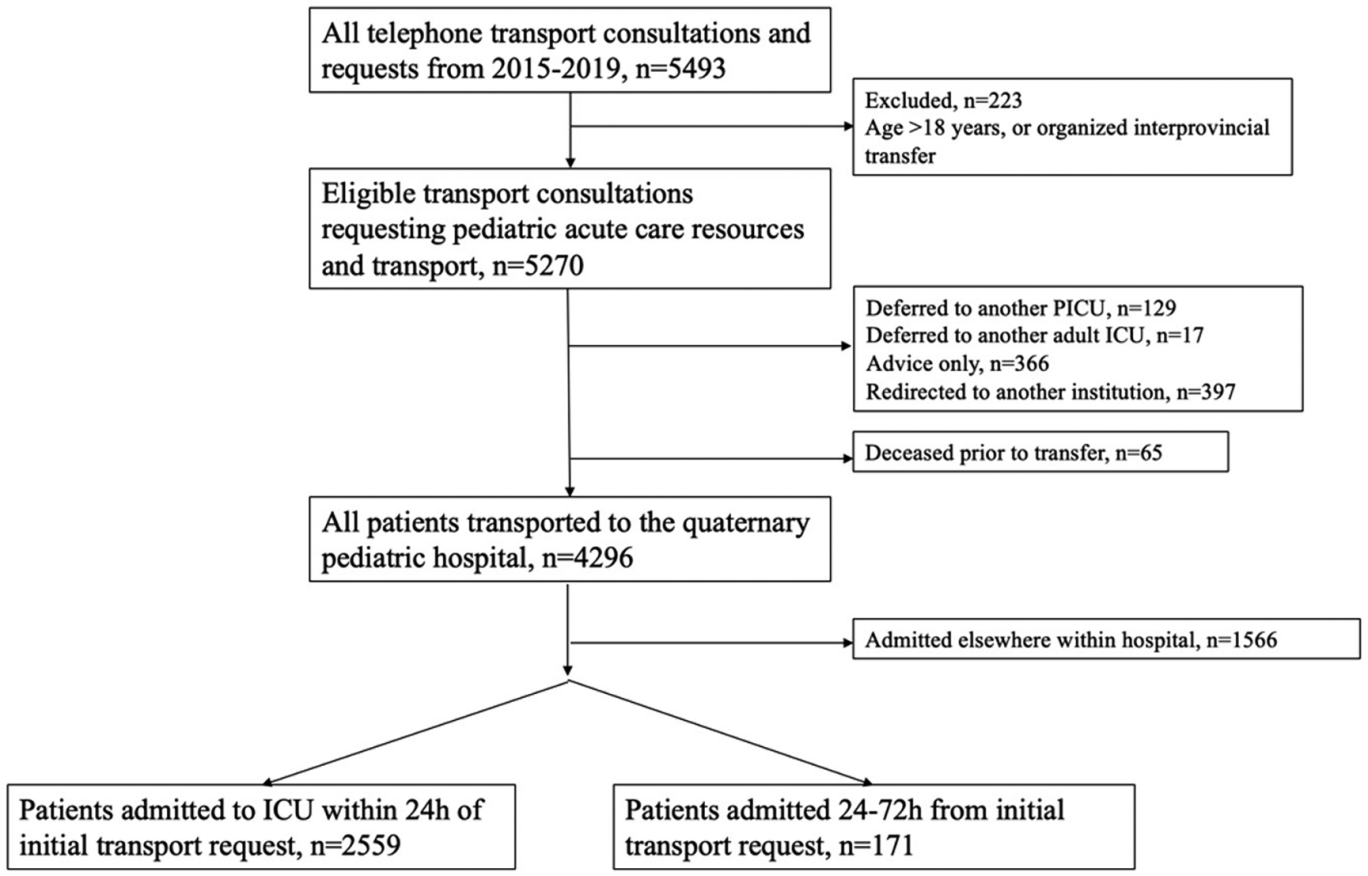
Flow diagram describing cohort of transported ICU patients from all transport requests during study period.

**Figure 2:**
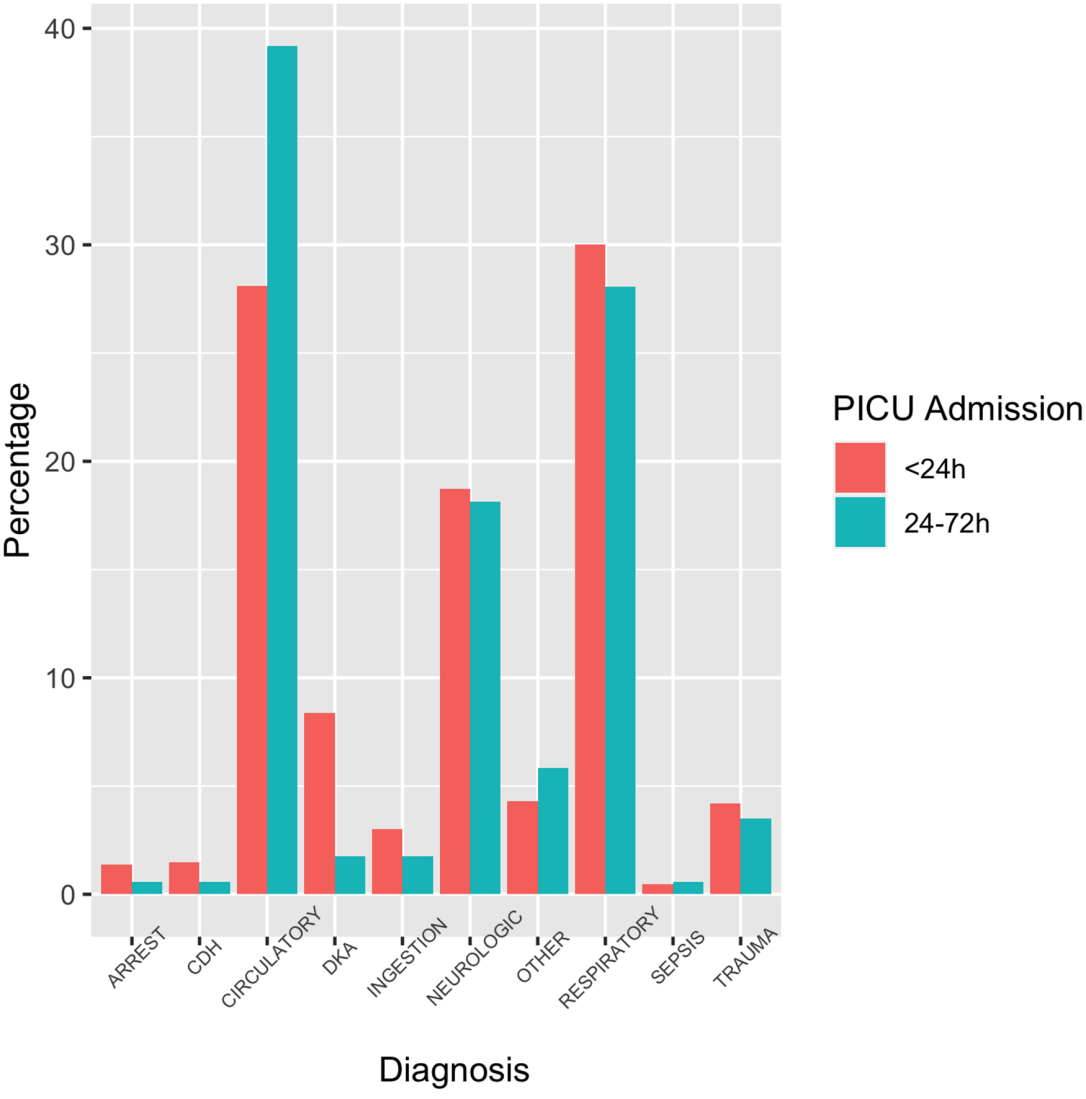
Primary diagnosis at the time of initial transfer request. Arrest is used as short form for cardiac arrest; CDH: congenital diaphragmatic hernia; Circulatory is used as short form for cardiac or circulatory; DKA: diabetic ketoacidosis; Ingestion is used for intentional or accidental ingestion/overdose.

A total of 2,730 patients were admitted after inter-facility transport to either the medical/surgical or cardiac ICU within 72 h of initial transport request over the 5-year period. Of these children, 2,559 (94%) were admitted within 24 h and 171 (6%) patients were admitted between 24 and 72 h from initial transport request (Figure 1). Children admitted between 24 and 72 h were younger and more likely to be from a rural centre, when compared to those admitted to the PICU within 24 h (Table 1). The median age of children that were admitted between 24 and 72 h was 7 months, and the most common diagnoses were cardiac (39%), respiratory (28%) and neurologic (18%) (Figure 2).

There was a small statistically significant difference in median PRISM-IV score between children admitted <24 h and those admitted between 24 and 72 h (median score 5 vs. 3, *p* = 0.047) (Table 2). Those admitted within 24–72 h had an increased need for non-invasive mechanical ventilation (*p* = 0.09) in the PICU. Duration for both non-invasive and invasive mechanical ventilation was similar for both groups, as were maximum PELOD-2 scores and ICU lengths of stay. Among all patients transported and admitted to the ICU, mortality was 5.5%. The crude mortality of those admitted within 24 h was 5.8% and the mortality amongst those admitted within 24–72 h of initial call was 1.8%. The mortality amongst transported ICU patients with cardiac or circulatory diagnoses was 6% (47/786),) and among those with a respiratory condition was 5% (38/816).

Over the 5-year study period, there were 5,493 unique calls to our transport and consultation telephone line, via CritiCall. Of all eligible transport calls, 82% (4,296/5,270) were ultimately brought to our quaternary care hospital, of which 60% (2,559/4,296) resulted in admission to the ICU within 24 h from the initial call. Of the remaining 1,737 patients, only 10% (171/1,737) were ultimately admitted to the PICU after a period of observation in another hospital setting.

The most common diagnosis reported for all telephone calls was respiratory (33%), followed by cardiac (24%) and neurologic (19%) conditions. Most phone calls and requests for transfer originated in hospitals with paediatric services (83%). For all patients brought to the quaternary care hospital, the median distance (as crow flies) was 28.1 km (15.1–43.8). The furthest distance by land was 1,697.2 km and the shortest distance was 0.23 km. A total of 65 patients died prior transfer. Among these deceased patients, the most common reported diagnosis was cardiac diagnosis (45%), followed by cardiac arrest (29%) and trauma (12%). Most of the deceased patients were from large urban centers (74%) and 8% were from rural or small urban communities.

In a subgroup analysis, over the 5-year study period, a total of 581 patients were ultimately admitted to the institution's cardiac ICU after inter-facility transport. Eighty-three percent of patients admitted to the cardiac ICU after transport were younger than 1 year of age at admission. Fifty-nine percent were male and the median distance from referring centre to our institution was 35.8 km (IQR: 18.8–87), as the crow flies. Among cardiac patients, 506 patients (87%) were from large urban centers and 9% were from rural or small urban centers. Ninety percent of patients admitted to the cardiac ICU were admitted within 24 h from initial phone call. The median PRISM-IV for all patients admitted to the cardiac ICU was 3 (0–7). Of these 581 patients, 379 (65%) required invasive mechanical ventilation for a median of 4 days (2–9) and the median ICU length of stay was 13 (7–27) days. The maximum PELOD-2 score during admission was 12 (10–21) and ICU mortality was 5.7% (33/581) among those transported to the cardiac ICU.

## 4 Discussion

Compared to children admitted within 24 h to the ICU at SickKids following inter-facility transport, those admitted to the ICU between 24 and 72 h following initial transport request were younger, were slightly less sick with a lower PRISM-IV score, and had a slightly greater need for non-invasive mechanical ventilation. Additionally, only approximately 10% of patients that were initially redirected to another care unit ultimately required PICU admission within 72 h from initial transport request. The proportion of children requiring mechanical ventilation and the PICU length of stay did not differ between the two groups. These results suggest that at the time of initial transfer request, patients who are sicker are being brought to the ICU directly and arrive within 24 h. Those who admitted after 24–72 h following initial call were likely accurately assessed to be less sick, possibly not in need of immediate critical care, and thus were redirected to another care setting before ultimately being admitted to the PICU.

Patients admitted to the PICU within 24–72 h after transport call were initially redirected to another care setting within The Hospital for Sick Children or observed at their referring center. The patients in this cohort were younger, residing in more rural settings, and a greater proportion of these children had cardiac diagnoses. These data suggests that at the initial telephone call, patients who may be perceived to be at increased risk of deterioration are possibly being transported to a quaternary care pediatric center for observation, where critical care can easily be accessed in an acute deterioration. This can be particularly true of patients residing in rural settings, where care escalation may be difficult or delayed, and in whom transfer may be preemptively organized regardless of patient severity. This is reflected in the characteristics of patients who were redirected and who did not require PICU (Supplementary Table S1). Given specialized pediatric transport teams are a limited resource, it is important to identify that characteristics including age and rurality can influence which children need to be transported for closer observation in a higher care setting. This is particularly important as transport is associated with financial and environmental consequences and can provide risk to the patient, and telemedicine might provide additional valuable information for patient assessment and disposition. Overall, only 10% of those patients observed in another care setting were ultimately admitted to the PICU. This suggests the initial triage decisions at the time of first call generally correctly identified patients in need of admission to PICU and those who could safely be observed. These results further suggest this initial triage did not result in delays in accessing critical care and subsequent increased length of stay, increased duration of invasive mechanical ventilation and increased mortality among those children initially redirected.

The overall ICU mortality for all transported patients was 5.5%. The mortality of those admitted within 24 h was significantly higher than the mortality among those who were initially redirected. The mortality amongst all transported patients is higher than overall PICU mortality, which is a consistent finding from other Canadian studies examining patients transported to PICUs (7, 9–11). The higher crude mortality rate amongst transported patients has also been reported in the UK, however after risk adjustment, the UK mortality rate was in fact lower for transported patients (20). This inconsistency highlights the need for further studies that examine how distance transported, mode of transport, composition of transport teams and pre-transport therapies may impact outcomes in transported patients in different countries. Given its large geographic catchment area, Canadian children transported to critical care units have different experiences than those in countries with a smaller geographic footprint. The required skills and need for transport systems in Canada are unique given the many remote and rural regions served, with the majority of pediatric tertiary care centers distributed in southern, large urban centres. This results in a high volume of long-distance transports of critically ill children for access to PICU level care.

This is the first description of children with a cardiac diagnosis admitted to a cardiac ICU following inter-facility transport in Canada. The majority of patients were under 1 year of age, were from urban centres, and were transported relatively short distances. The PRISM-IV score was not significantly different in this subgroup, nor was their overall crude mortality rate.

### 4.1 Limitations

There are several limitations to this study. First, data pertaining to the initial presentation of patients and specific diagnosis were limited, owing to the retrospective nature of this study and the limited data variables in the existing database. Additionally, the database did not describe the mode of transport or the duration of transport. The lack of recorded time from initial call to transport team arrival and the duration of transport limited our ability to interpret the effects of transport time on patient outcomes. Data were not available regarding management and evolution of children between initial call and admission to the ICU. This limits our ability to understand how children who were admitted within 24–72 h may have evolved between initial call and eventual admission. Knowing where they were initially admitted and managed would be beneficial when making triage decisions for transport. Only 79% of participants had complete data. The characteristics of patients with missing outcome data were reviewed and the data were felt to be missing at random. Lastly, transport distances were estimated based on distances from listed addresses of the care facilities where the call originated to the final destination of The Hospital for Sick Children. While this method has been used in several other studies, it may not accurately reflect the transport distance or duration, given the many components of transfer including driving, flying and wait times in airports (7, 9). Moreover, while children presenting to rural hospitals were assumed to be from rural locations, we did not have access patient's home addresses to confirm this assumption.

### 4.2 Conclusions

Compared to children admitted within 24 h to the ICU following inter-facility transport, those admitted between 24 and 72 h following initial transport request were younger, had a lower PRISM-IV score and had a greater need for non-invasive mechanical ventilation. Additionally, those admitted within 24 h following transport had a higher mortality rate than those admitted within 24–72 h. Following transport and observation in another hospital setting, only 10% of patients were ultimately admitted to the PICU. This study highlights the need to better understand which factors influence disposition decision making at the time of initial transport request. Given the vast resources used to train and employ specialized pediatric transport teams, and to complete a pediatric critical care transport, it is important to better understand which patient and setting characteristics may be used to safely triage children at time of initial critical care call request. Further research should focus on the impact of distance, transport team composition, duration of transport, and mode of transport on illness severity and risk of mortality. Moreover, given the vast geography of Canada, additional research is needed into the impact of both transport and redirection to a non-ICU care setting on patients and their families, as well as the environmental footprint of interfacility transport.

## Data Availability

The data analyzed in this study is subject to the following licenses/restrictions: Datasets belonging to The Hospital for Sick Children, Department of Critical Care Medicine. Restricted to those with approval to access data from the Hospital for Sick Children Research Ethics Review Board. Data are available upon request. Requests to access these datasets should be directed to Christina Maratta, christina.maratta@mcgill.ca.

## Footnotes

^1^ https://www12.statcan.gc.ca/census-recensement/2016/ref/dict/geo049a-eng.cfm
