# Corrigendum: Direct vs. redirected admission of critically ill children to PICU after interfacility transfer: a retrospective cohort study

**DOI:** 10.3389/fped.2024.1399382

**Published:** 2024-03-21

**Authors:** C. Halgren, G. M. Annich, C. Maratta

**Affiliations:** ^1^ Department of Critical Care, Hospital for Sick Children, Toronto, ON, Canada; ^2^ Interdepartmental Division of Critical Care Medicine, University of Toronto, Toronto, ON, Canada; ^3^ Institute of Health Policy, Management and Evaluation, University of Toronto, Toronto, ON, Canada; ^4^ Department of Paediatrics, University of Toronto, Toronto, ON, Canada

**Keywords:** pediatric transport, critical care transport, interfacility transport, PICU, remoteness

A Corrigendum on Direct vs. redirected admission of critically ill children to PICU after interfacility transfer: a retrospective cohort study By Halgren C, Annich GM and Maratta, C (2024). Front Pediatr. 12:1307565. doi: 10.3389/fped.2024.1307565

In the published article, Shayanthan Parameswaran, Norbert Chin, Mohit Ramnani, Rachelle Crane, Eva Ta were left out of the Acknowledgement section of the manuscript.

This sentence previously stated:

“We thank Dr. Christopher Parshuram, Dr. Haifa Mtaweh and Dr. Melany Gaetani for their thoughtful discussion, feedback and support.”

The corrected sentence appears below:

“We thank Shayanthan Parameswaran, Norbert Chin, Mohit Ramnani, Rachelle Crane, Eva Ta, Dr. Christopher Parshuram, Dr. Haifa Mtaweh and Dr. Melany Gaetani for their help, support and thoughtful discussion.”

The authors apologize for this error and state that this does not change the scientific conclusions of the article in any way. The original article has been updated.

