# Supplementary material for: Direct vs. redirected admission of critically ill children to PICU after interfacility transfer: a retrospective cohort study
Source: Front Pediatr. 2024 Feb 16;12:1307565. doi: 10.3389/fped.2024.1307565 (PMC10904567; doi:10.3389/fped.2024.1307565)
Supplement: Supplementary file 1 [file Table1.docx]

Supplemental Table 1: Characteristics of all redirected patients after inter-facility transport

| **Characteristic** | **All redirected,**  **n= 1,737** | **Admitted to PICU within 24-72h from call, n=171** | **Not admitted to PICU within 72h of call,**  **n= 1,566** |
| --- | --- | --- | --- |
| Age in years, (IQR) | 2.7 (0.7-8.2) | 0.6 (0.03-5.7) | 2.9 (0.8-8.4) |
| Primary Diagnosis, n (%)  Cardiac/Circulation  Cardiac Arrest / VSA  CDH  DKA  Ingestion/Overdose  Neurologic  Respiratory  Sepsis/Infection  Trauma/Drowning/Burn  Other  Missing | 301 (17.4)  3 (0.2)  1 (0.1)  19 (1.1)  52 (3)  375 (21.6)  626 (36.1)  21 (1.2)  118 (6.8)  217 (12.5)  4 (0.2) | 67 (39.2)  1 (0.6)  1 (0.6)  3 (1.8)  3 (1.8)  31 (18.1)  48 (28.1)  1 (0.6)  6 (3.5)  10 (5.9)  0 | 234 (15)  2 (0.1)  0  16 (1)  49 (3.1)  344 (22)  578 (37)  20 (1.3)  112 (7.2)  207 (13.3)  4 (0.3) |
| Urban Centre*, n (%)  Rural or small  Medium  Large  Missing | 186 (10.7)  165 (9.5)  1362 (78.4)  24 (1.4) | 31 (18.1)  0  149 (81.9)  0 | 155 (9.9)  165 (10.5)  1222 (78)  24 (1.5) |
| Distance**, (minimum, maximum) | 23.6 (0.15–346.3) | 41.5 (25.6–79.3) | 12.5 (0.15–346.3) |

Abbreviations: CDH: congenital diaphragmatic hernia; DKA: diabetic ketoacidosis; VSA: vital signs absent; IQR: interquartile range.

All continuous data are presented in medians and interquartile ranges, unless otherwise specified.

* Rural/Small center: any area outside of a population center or a population between <1000 and 29,999. Medium center: population between 30,000 and 99,999. Large center: population of 100,000 or more.

**Distance from referral center to the institution in kilometers (as the crow flies). The IQR for 23.6 was (7.9, 41.5), for 41.5 was (41.5, 41.5) and for 12.5 was (4.5, 34.6).
